# Preregistration of Analyses of Preexisting Data

**DOI:** 10.5334/pb.493

**Published:** 2019-08-22

**Authors:** Gaëtan Mertens, Angelos-Miltiadis Krypotos

**Affiliations:** 1 Department of Clinical Psychology, Utrecht University, Utrecht, NL; 2 Leuven Research Group on Health Psychology, KU Leuven, Leuven, BE

**Keywords:** Replicability, Transparency, Open Science, Archival Data

## Abstract

The preregistration of a study’s hypotheses, methods, and data-analyses steps is becoming a popular psychological research practice. To date, most of the discussion on study preregistration has focused on the preregistration of studies that include the collection of original data. However, much of the research in psychology relies on the (re-)analysis of preexisting data. Importantly, this type of studies is different from original studies as researchers cannot change major aspects of the study (e.g., experimental manipulations, sample size). Here, we provide arguments as to why it is useful to preregister analyses of preexisting data, discuss practical considerations, consider potential concerns, and introduce a preregistration template tailored for studies focused on the analyses of preexisting data. We argue that the preregistration of hypotheses and data-analyses for analyses of preexisting data is an important step towards more transparent psychological research.

## Introduction

Cumulative research requires that research findings are replicable (i.e., repetition of a study’s results using similar procedures as the original study; Brandt et al., 2014; Goodman, Fanelli, & Ioannidis, 2016).^1^ Replicable findings can help cumulative research by building on reliable observations and expanding a body of knowledge, thereby resulting in theoretical development, innovation, and evidence-based solutions for societal problems. In contrast, non-replicable findings may not always be useful for scientific progress. Although a non-replicable result could help in determining the conditions under which a finding can/cannot be replicated, if the majority of research cannot be replicated due to poor, or questionable, research practices, then the corresponding results may lead to fruitless research projects, stall theoretical development, and, if undetected, result in unreliable and possibly harmful applications. Alas, recent findings indicate that replicability in psychology is at alarmingly low levels, with many influential effects not being replicated, although the appropriate methodology and adequate sample sizes were used (Klein et al., 2014; Open Science Collaboration, 2015).

Several reasons for the limited replicability of findings in the psychology literature have been suggested, including the use of underpowered studies (Bakker, van Dijk, & Wicherts, 2012; Schimmack, 2012), inadequate inferences from statistical tests (Wagenmakers, Wetzels, Borsboom, & van der Maas, 2011), a lack of a unified theoretical framework (Muthukrishna & Henrich, 2019), and problematic incentives (Lilienfeld, 2017). From the different reasons offered, we focus on two issues in particular to highlight the need for preregistration of research plans. One is post-hoc hypothesizing and analyses. This refers to the determination of the research hypotheses and/or the statistical analyses of a study based on the direction of the results rather than on sticking to the a priori hypothesis and/or the planned statistical analyses, while presenting those post-hoc decisions as specified a priori. These questionable research practices can dramatically inflate the number of false-positives (because with sufficient tests, statistically “significant” effects will always be found and there is an infinite number of possible post-hoc explanations for such findings; Kerr, 1998; Simmons, Nelson, & Simonsohn, 2011). Another reason is publication bias, or in other words the tendency to publish results that support the existence, rather than absence, of an effect. Publication bias is deleterious for science as it also inflates the number of false-positive findings in the literature and gives an incorrect picture about the robustness of an effect (Easterbrook, Gopalan, Berlin, & Matthews, 1991; Franco, Malhotra, & Simonovits, 2014).

Preregistration of research plans can help to (partly) prevent these problematic research practices (Asendorpf et al., 2013; Munafò et al., 2017; Nosek et al., 2015; van ’t Veer & Giner-Sorolla, 2016; Weston, Ritchie, Rohrer, & Przybylski, 2019). Preregistration refers to the specification of a study’s hypotheses, methodology, and statistical analyses before inspecting the research data. Preregistration takes typically the form of a document that is made publicly available on a timestamped repository or website (e.g., osf.io or clinicaltrials.gov). Since the hypotheses, methods, and statistical plan are known before the beginning of a study, the chances of presenting post-hoc hypothesizing and analyses as a priori decisions are reduced. Furthermore, in case of registered reports (see below), such transparent research practices may also lead to higher chances of acceptance by a journal, as the authors can prove that all their analytical plans were determined a priori, protecting the authors from rejection based on negative findings (Allen & Mehler, 2019). Increasingly, journals and funding agencies are encouraging and requiring that researchers preregister their plans for studies (see for instance the preregistration badges offered by journals such as Psychological Science, Kidwell et al., 2016).

Although preregistration is nowadays common for original data sets (i.e., data sets that still have to be collected), with several templates being available already (van ’t Veer & Giner-Sorolla, 2016; Veldkamp et al., 2018), there has been relatively little attention for the reasons why such preregistration may be applicable to also the analysis of preexisting data (i.e., data that have been collected and/or published before). Importantly, this type of research differs fundamentally from research in which original data are collected as many important decisions (e.g., the followed methodology and the sample size) have already been made. As analyses of preexisting data are a crucial tool for the field of psychology, it is important to discuss the need for preregistration of such studies and establish appropriate guidelines (Syed & Donnellan, 2018; Weston & Bakker, 2018; Weston et al., 2019).

The goal of this paper is to provide concrete arguments as to why preregistration of analyses of preexisting data is important and helpful. To also enable the easy preregistration of such studies, we provide a template for preregistration of analyses of preexisting data. Because there are several different types of re-analysis of preexisting data – e.g., analyses of (a combination of) existing databases (e.g., the European Social Survey, genetic databases, national election results), re-analysis of existing studies, a combined analysis of different studies (mega-analysis), analysis of simulated data, computational modelling, and meta-analyses – we aim to provide general guidelines for all different types of such analyses. However, particularly for meta-analyses, it should be noted that extensive guidelines are available already (Lakens, Hilgard, & Staaks, 2016; Moher et al., 2015).

## Arguments for Preregistration of Analyses of Preexisting data

Below we summarize why it is useful to preregister analyses of preexisting data. Please note that although some of the reasons are similar to the reasons for preregistrating of an original study, here we focus on the reasons for preregistration of analyses of preexisting data specifically.

### Distinguishing between exploratory and confirmatory research

Empirical research can broadly entail two different epistemic goals: exploration and confirmation (De Groot, 1969). Particularly, exploratory research is often hypothesis generating and curiosity driven: new ideas and theories can develop on the basis of collected data and/or exploratory data analyses. Confirmatory research, on the other hand, involves testing specific predictions (hypotheses) derived from theories. Both exploratory and confirmatory research serve important functions for science. However, these two types of research should be distinguished. More specifically, much of the research in psychology is concerned with testing hypotheses. However, a problematic practice in which researchers often engage is to first explore the data and then formulate hypotheses that correspond with the obtained results, without clarifying this order or even actively distorting the order of operations (Kerr, 1998; Simmons et al., 2011). This practice invalidates the commonly used statistical procedures in the null-hypothesis testing framework, because the generation of hypotheses and testing of hypotheses are no longer independent (i.e., the formulation of the hypothesis and the ‘test’ of the hypothesis are based on exactly the same data), inflating the number of false-positive results (De Groot, 2014; Simmons et al., 2011; Wagenmakers, Wetzels, Borsboom, van der Maas, & Kievit, 2012). To prevent such mischaracterization of the evaluation of hypotheses, a distinction should be made between exploratory (hypothesis-generating) and confirmatory (hypothesis-testing) research (Jebb, Parrigon, & Woo, 2017; Simmons et al., 2011; Wagenmakers et al., 2012).

The distinction between these different types of research is particularly straightforward for the analysis of original data sets: Hypotheses can be specified and preregistered before any data is collected. Hypothesis and data-analysis steps that were in place before data collection can be seen as confirmatory, and any additional analyses that were unplanned and based on the inspection of the data can be seen as exploratory. This distinction can be more difficult, though, for analyses of preexisting data because at least part of the data is known already. This difficulty in definition, however, should not necessarily withhold researchers from preregistrating their analyses of preexisting data. On the contrary, we think that researchers interested in conducting confirmatory research on preexisting data can greatly benefit from using preregistrations to help establish the confirmatory nature of their research.

Although confirmatory research can be difficult using analyses of preexisting data (because data patterns are often known), there are conditions when such analyses could be considered largely confirmatory. This is the case, for example, in case of large data sets where multiple variables have been collected. In such cases, researchers could preregister their plan, and only afterwards (request) access and analyze the data (for an example see Hussey et al., 2019). We provide more examples of how the confirmatory nature of the research can be ensured for analyses of preexisting data in the next section (‘Practical Considerations for the preregistration of analyses of preexisting data’). Please note, however, that even if a single *relevant* variable has been analyzed before, the authors have prior knowledge of (parts) of the data-patterns already. Therefore, analyses of datasets that have been explored before should be, strictly speaking, categorized as exploratory.

Finally, not only in the research and writing process the distinction between confirmatory and exploratory research is sometimes lost, but also in the publication process. That is, journal editors and reviewers sometimes request additional analyses from researchers without necessarily requiring researchers to report these additional analyses as exploratory, ask that authors change their hypotheses to fit the observed results, or ask authors to simplify or disregard results that do not fit the initial hypothesis (Maner, 2014). These practices by reviewers and editors also obscure the distinction between exploratory and confirmatory research. Preregistered data-analysis plans can protect the researchers against such requests or allow readers from scientific journals to retrieve the preregistrations and judge for themselves what part of the research were confirmatory and exploratory.

### Protection against publication bias

It is a well-known problem that studies that report significant results have higher chances of getting accepted for publication by a scientific journal, compared to studies that report non-significant results (Coursol & Wagner, 1986; Levine, Asada, & Carpenter, 2009; Rosenthal, 1979). Such publication biases are apparent from studies showing that the number of positive results reported in psychology journals exceeds 90% (Bakker et al., 2012; Fanelli, 2010), despite the power of the studies being commonly low (i.e., on average .23, .60, and .78 to detect small, medium, and large effect sizes, respectively; Szucs & Ioannidis, 2017). These numbers demonstrate the high probability of false-positive results being reported in the literature.

There are ways in which preregistration of analyses of preexisting data can prevent publication bias. This is the case of registered reports (also called *reviewed preregistration*; van ’t Veer & Giner-Sorolla, 2016). Originally developed in the area of clinical trials (see Dickersin & Drummond, 2003) and more recently further expanded to research in psychological science (Chambers, Feredoes, Muthukumaraswamy, & Etchells, 2014), registered reports refer to the reviewing of the introduction and the methodology of a study *before* the study is conducted. Upon acceptance of the research plan, journals typically commit to publishing the registered study regardless of the results of the study, provided that there were no (major) deviations of the research plan. Recent results do indeed indicate that registered reports result in a higher percentage of non-significant findings being published, compared to the traditional publishing format (see Allen & Mehler, 2019).

We believe that registered report format could also be used for analyses of preexisting data. Particularly, registered reports for analyses of preexisting data could include the statistical analysis plan together with arguments as to why the specific data set can help in answering the hypotheses the authors want to answer (see our template below for more specific information of what should be documented for analyses of preexisting data). Such, registered reports can help authors to publish their analyses of preexisting data, even when the analyses do not confirm the authors’ initial hypothesis.

Although not all journals publish registered reports, the list of supporting journal is increasing (see https://cos.io/rr/ for an up-to-date list). When a preferred outlet does not provide the option to publish registered reports, authors could preregister their study in the traditional (*unreviewed preregistration*; van ’t Veer & Giner-Sorolla, 2016) way and provide evidence as to their being blind to the data before the beginning of the data analyses. Although such a plan does not ensure the publication of the study, it does provide a transparent way to convince editors and reviewers that the analyses plan was decided before inspecting a study’s result.

### Countering researchers’ degrees of freedom in data analysis (overfitting, cherry-picking, variable swapping, flexible model selection, subsampling)

Data analyses usually entails many different steps (cleaning of the data, selecting a statistical model, selecting variables and covariates) in which researchers have to make decisions. These decisions (also known as researchers’ degrees of freedom; Simmons et al., 2011) can influence the final results. To illustrate, recent studies suggest that the use of different analytic procedures for answering a research question, even when the same dataset and similar flavors of the same model are used (e.g., drift diffusion model, logistic regression), sometimes results in different parameters estimation, and even different directions of results (Boehm et al., 2018; Dutilh et al., 2018; Silberzahn et al., 2018).

It could be argued that analyses of preexisting data, similar to analyses of original data, have a high chance of false-positive results. For example, when dealing with a large data set it could be tempting to include some extra variables in the analysis for, potentially, explaining more variance in the data. This, however, introduces the problem of overfitting a model to the data, and as such limits the generalizability of the model to new data (Lee et al., 2019). On the other hand, someone could be tempted to cherry-pick the variables that better fit their hypothesis, and ignore variables that contradict it, something that can result in an imprecise picture of the effect (Lewandowsky & Bishop, 2016). Another problem is that large datasets, on which analyses of preexisting data are often conducted, usually contain a wide range of variables. Researchers may be tempted to switch variables or the statistical model when the original hypotheses does not hold up. Again, such a data-driven approach can hamper the generalizability of results given that it inflates the chances of observing spurious results. Finally, researchers could decide to exclude the data from several participants, data clusters or time points because the results do not fit well with their hypothesis. Again, unspecified decisions allow to capitalize on chance and inflate the probability of finding spurious results (Murayama, Pekrun, & Fiedler, 2014).

Such potential variability in the result outcomes, even with the same data set, should encourage researchers to fully preregister their analyses, even for exploratory purposes, as this could provide a safety-net against overfitting their models, cherry-picking variables, variable switching, flexibility in model selection, and sub-sampling parts of the dataset. Of course, a preregistration does not prevent a researcher from selecting the most appropriate statistical tools when this was not included in the preregistration. However, it does clarify for the researcher and readers that this decision was made post-hoc rather than a priori.

## Practical Considerations for the preregistration of analyses of preexisting data

There are a number of practical considerations that pose a considerable challenge for the preregistration of analyses of preexisting data. Below, we consider such challenges and suggest concrete solutions.

### Ensuring preregistration before data analyses

An important challenge for preregistration of analyses of preexisting data is that the data are already available. Therefore, timestamps included in the data files cannot be used to determine whether the data-analysis plans were specified before analyzing the data. This is an important issue because without timestamping, researchers can easily antedate their data-analysis plans while, in fact, data-analysis were not conducted independent of data-evaluation. In this case, researchers and the scientific literature suffer again from the same biases and limitations mentioned previously. There are, however, ways to reduce this risk.

If the data are not available to the researcher prior to the beginning of the analyses, when for example the data are available only in a private server, researchers can ask for documentation from the administrators of the original data sets, specifying when the data were made available for the analyses. If the data were available to the researchers before the preregistration, researchers could provide an online supplement specifying when the data were acquired, when the preregistration was done, and when the analyses mentioned in the preregistration were conducted (see our recommendations below).

Additionally, researchers could also provide simulated data and data-analysis syntax to demonstrate that the data-analysis pipeline was already in place before the actual analyses were conducted. These materials (i.e., simulated data and data-analysis syntax) could be submitted to a journal as a registered report and/or to administrators of a database to demonstrate that the hypotheses and the planned data-analysis pipeline were in place before the data was accessed (for a recent project using such a work-flow in the context of attitude research see Hussey et al., 2019).

Another option is to collaborate with independent research groups or data-analysis consultants who can conduct the analyses independent of the researchers who came up with the original idea. As such, data-evaluation may be achieved independently from hypothesis construction, though it should be ensured that there are no financial ties or other dependencies between the two groups that may compromise the independent analysis and evaluation of the data.

Finally, this point is also important for administrators of databases and individual researchers. It may be useful, in case the data are not made available already, to require researchers to provide a detailed pre-registration of their hypothesis and data-analysis plans before giving them access to a database or before sharing datafiles. However, we do want to highlight that we encourage the open sharing of data and materials (see Krypotos, Klugkist, Mertens, & Engelhard, 2019). We merely mention this option for consideration in certain situations (e.g., when conducting analyses on sensitive data that are not publicly available, such as patient records) and to encourage researchers requesting data to be precise and transparent about their aims and hypotheses when planning their analyses of preexisting data.

### Exploration, confirmation, and prediction in analyses of preexisting data

It has been recently suggested that science is inherently complicated, with multiple processes being involved, making it difficult to isolate a single effect. Rather than establishing whether or not an effect replicates, we should be better off exploring which effect occurs under which conditions (Shiffrin, 2019) or focusing on our ability to maximally predict, rather than causally explain, human behavior (Yarkoni & Westfall, 2017). Even in such cases preregistration can be useful. Particularly, in the case of explorative research, observations often have to eventually be confirmed on independent data to ensure that the observations also generalize to different datasets with different contextual factors that may influence the relevance of moderators. Likewise, researchers interested in model building (computational and predictive modeling) also need to verify that their models apply to different datasets than on the data on which their models were originally developed (Lee et al., 2019). In both cases, the confirmatory work can profit from preregistration to avoid that the interpretation of the findings is influenced by post-hoc hypothesizing or overfitting of a model to the data.

In addition, even purely explorative research or model development commonly relies on certain assumptions or prior observations (e.g., independence of datapoints, included variables were reliably measured). It may be good practice to provide information about such assumptions and background of the data for purely exploratory analyses and modelling as well in order to increase transparency. At the very least, it is important to specify the way in which the results will be evaluated (e.g., which fit indices will be used; what are the evaluation cut-offs; corrections for multiple testing), because different ways of evaluating the results can lead to different conclusions (Lee et al., 2019). A preregistration plan can help researchers with specifying these details of their exploratory and modelling work (Lindsay, Simons, & Lilienfeld, 2016). Once again, we would like to highlight, as also other have done (Lindsay et al., 2016; Munafò et al., 2017; Simmons et al., 2011), that preregistration does not exclude unplanned work. It merely makes the choices made by researchers more transparent.

## Potential criticisms and concerns

Despite the advantages of preregistration, there are some potential concerns that merit some discussion. We address a number of these concerns pertaining to analyses of preregistration of analyses of existing data specifically here. For a consideration of concerns about preregistration more generally (i.e., also including preregistration of original data collection) see Allen and Mehler (2019) and Lindsay (2019).

First, even if preexisting data are readily available, they may lack proper documentation that could allow researchers to easily understand the structure of the data (e.g., what 0 and 1 mean in a column named ‘gender’). This problem has been acknowledged in the literature and that is why researchers are now urged to use make their data be Findable, Accessible, Interoperable, and Re-Usable (FAIR) (e.g., Wilkinson et al., 2016). We have also acknowledged this problem before and we have described concrete steps, as well developed the relevant software, for better archiving research data (Krypotos et al., 2019).

Second, researchers should be aware that preregistration does not automatically mean that the preregistered analytical framework was appropriate and the results cannot be refuted. To illustrate, even if a researcher thinks that a specific statistical model is the right one to answer a specific question, that does not mean that other plausible, and even better, models do not exist that could potentially lead to different results (e.g., Boehm et al., 2018; Dutilh et al., 2018). In such cases, it could be worthwhile for researchers to consider alternative plausible analyses and test how the direction of results may depend from the used statistical models (see multiverse analysis in Steegen, Tuerlinckx, Gelman, & Vanpaemel, 2016). If conflicting outcomes arise, the researcher should be concerned about the robustness of the results. Another approach that could be considered is to fine-tune and validate the data-analysis steps first on part of the data (i.e., exploratory) and then use the remaining data for confirmatory analyses. Such exploratory analyses of a subset of the data can be part of the preregistration (see Q8 below).

Third, researchers may object that preregistration of analyses of preexisting data require a substantial time investment, interfering with the researchers’ limited research time, and that it hampers the rapid development of science. We see several important arguments against this concern. First, given the developed awareness regarding the replication crisis in psychology and the rapid developments of services to upload and share data and research materials, research findings that were not clearly preregistered may become more difficult to publish in the future. In fact, several universities, funding agencies and journals are taking steps to require the preregistration of research plans (e.g., for the Replication Studies grant of the Dutch Research Council, preregistration of planned studies in a database or repository is mandatory). Hence, rather than interfering with career prospects, proper research practices, including preregistration, will become an indispensable component of research and research careers (see also Allen & Mehler, 2019). Second, rather than a waste of time, more replicable research will actually save time for the research community by no longer having to invest in trying to replicate spurious findings. Instead, it will become possible to properly invest time and resources for scientific research into more robust rather than spurious findings. Third, determining hypotheses and data-analyses steps can actually streamline the research process. In fact, all steps within a preregistration are necessary steps that have to be done anyway in any research project. Preregistration only requires researchers’ to specify this *before* conducting the analyses, rather than afterwards. Furthermore, though writing preregistrations and compiling data-analyses syntax may require a substantial time investment at first, it can facilitate subsequent research by having an appropriate rationale and data-syntax in place. Finally, having a clearly specified rationale for certain data-analysis steps can protect against requests for unnecessary exploratory analyses by journal reviewers and editors (Wagenmakers & Dutilh, 2016). In this way, again, preregistration can protect researchers from time-consuming exploratory analyses that may produce spurious results and that can obscure the original hypothesis of the planned research.

## Template proposal

Based on existing preregistration templates for new data collection (van ’t Veer & Giner-Sorolla, 2016) and the practical consideration we have reviewed above, we have developed a template for the preregistration of analyses of preexisting data (see Table 1). Our template is inspired by the format of aspredicted.org, in which a number of questions have answered detailing the planned research (focused primarily on original experimental research). We have formulated 10 questions that are intended to provide maximal transparency about the steps undertaken in the evaluation of hypotheses using analyses of preexisting data. We suggest that researchers answer these questions in a document and make this available through an online and preferably time-stamped repository (such as osf.io). Alternatively, researchers could make use of the analyses of preexisting data preregistration template we provide in the *pss* software package (Krypotos et al., 2019). We briefly discuss each of the questions here and illustrate it with an example from our own research area (i.e., fear conditioning research).

**Table 1:** Template questions for the preregistration of analyses of preexisting data.

Question		Description

1.	What is the hypothesis that will be investigated?	*Provide a brief description of the relevant theory and formulize the hypothesis as precisely as possible*.
2.	How will the crucial variables be operationalized?	*State exactly how the variables specified in the hypothesis will be measured*.
3.	What is the source of the data included in the analyses?	*Specify the source of the obtained data. Also provide information about the context of the data source and clarify whether the data has been previously published. In case of simulated data, provide information on how the data was generated*.
4.	How will this data be obtained?	*Specify how the data will be requested or accessed. Clarify whether the data were already available and whether the dataset has been previously explored or analyzed*.
5.	Are there any exclusion criteria for the data?	*Specify whether there were any criteria for the exclusions of certain datasets, observations or time points*.
6.	What are the planned statistical analyses?	*Specify the statistical model that will be used to analyze the data and describe the data pre-processing steps. Be as specific as possible and avoid ambiguity*.
7.	What are the criteria for confirming and disconfirming the hypotheses?	*Specify exactly how the hypothesis will be evaluated. Give specific criteria relevant to the used analytical model and framework (e.g., alpha-values, Bayes Factor, RMSEA)*.
8.	Have the analyses been validated on a subset of the data? If yes, please specify and provide the relevant files.	*Indicate whether the proposed data-analyses have previously been validated on a subset of the data or a simulated dataset. If so, provide the data files and data syntax*.
9.	What is known about the data that could be relevant for the tested hypotheses?	*Please describe any prior knowledge that you have about the data set (e.g., the known mean of a variable) that is relevant for your research question*.
10.	Please provide a brief timeline for the different steps in the preregistration.	*Provide the (foreseen) dates for the different steps in this preregistration form*.

**Q1: What is the hypothesis which will be investigated?**

Provide a short description of the relevant theory and prior research, and specify the hypothesis as precisely as possible. For example: *We want to test whether discriminatory fear conditioning (i.e., stronger fear responses to a conditioned stimulus [CS*+*] paired with an aversive shock than to a stimulus not paired with an aversive shock [CS–]) is positively related anxious personality traits (see Indovina, Robbins, Núñez-Elizalde, Dunn, & Bishop, 2011)*.

**Q2: How will the crucial variables be operationalized?**

Specify how the variables mentioned in the hypothesis will be operationalized. Again, be as specific as possible to counter the possibility of variables-swapping or ambiguity for readers, reviewers, and editors. For example: *Anxious personality will be measured using the Trait version of the State-Trait Anxiety Inventory (Spielberger, Gorsuch, & Lushene, 1970). Cue discrimination will be measured as the difference in skin conductance responding (in μS) between CS*+ *and CS–*.

**Q3: What is the source of the data included in the analyses?**

Specify the source of the data. If necessary, provide some additional background, such as about the managing organization, other variables in the dataset, and potential bias in the dataset. In case of simulated data, provide details on how the data were generated. In case of re-analysis of previously published data, clearly refer to all publications with this data. For example: *The above-mentioned hypothesis will be evaluated on two previously published data sets (Krypotos, Arnaudova, Effting, Kindt, & Beckers, 2015; Mertens et al., 2016) and the available relevant data sets by researchers X, Y, and Z*.

**Q4: How will this data be obtained?**

Specify how the data will be requested and accessed. In case the preregistration was part of the requirements to access the data, mention this explicitly. In case the data is already available, mention this explicitly and describe in sufficient detail which parts of the data have been used before (see Q3). For example: *Researchers X, Y and Z working on fear conditioning will be contacted to share data sets regarding fear conditioning including both skin conductance responses and trait anxiety. Two other data sets are already available from our lab and have previously already been published (see Krypotos et al., 2015; Mertens et al., 2016) with a focus on unrelated topics (i.e., the role of instructions in fear conditioning and approach-avoidance training in fear conditioning)*.

**Q5: Are there any exclusion criteria for the data?**

Specify whether any datasets, observations, or timepoints that are potentially relevant will be excluded. Also clarify the reason(s) for these exclusions. For example: *Studies focusing exclusively on instructed fear conditioning (e.g., Mertens & De Houwer, 2016) will be excluded from our analyses, due to the fact that learning mechanisms may potentially be different in instructed and uninstructed fear conditioning (Tabbert et al., 2011)*.

**Q6: What are the planned statistical analyses?**

Provide specifics about the planned statistical analyses and avoid ambiguity (e.g., do not merely state that an ANOVA will be conducted if in fact a repeated measures ANOVA will be conducted). Give information about possible outlier exclusions or other data preprocessing steps. For example: *The different obtained datasets will be combined and a Pearson’s correlation will be calculated between the difference in skin conductance responses for CS*+ *and CS– on the last trial of the conditioning phase and trait anxiety scores. Skin conductance responses were divided by an individuals’ maximal response to account for inter-individual differences and were square-root transformed to normalize the distribution of the data (Dawson, Schell, & Filion, 2007)*.

**Q7: What are the criteria for confirming and disconfirming the hypotheses?**

Provide clear criteria for the evaluation of the hypotheses. In the Null-Hypothesis Testing framework this is usually the alpha-value and in a Bayesian Hypothesis Testing framework specifying what constitutes strong evidence for (dis-)confirming a research hypothesis. In case of statistical modelling, specify which criterium will be used for model fitting (e.g., Bayesian Information Criterion, Akaike Information Criterion, Root Mean Square Error of Approximation). For example: *We expect a significant (p* < *.05) correlation between discriminatory fear conditioning and trait anxiety scores*.

**Q8: Have the analyses been validated on a subset of the data? If yes, please specify and provide the relevant data-analyses syntax.**

Provide the necessary information if the analyses have previously been validated on a subset of the data (e.g., how many cases, what percentage of the dataset, which variables). Furthermore, provide the data-analysis syntax for evaluation by peers, reviewers, and editors. For example: *The planned statistical model was first evaluated on the datasets available from our lab (see data.sav, syntax.sps, output.spv). These results will be verified on the aggregated data of our own lab and the data sets made available by researchers X, Y, and Z*.

**Q9: What is known about the data that could be relevant for the tested hypotheses?**

Since knowledge about the data set could be available in advance (e.g., the mean values of some of the variables have been published already), it is desirable that the researcher(s) mention this prior knowledge in the preregistration template. This step ensures that the authors have shared what is known already, something that allows the readers to know what new information is original and which is not. For example: *In the original studies, significant discriminatory fear conditioning (i.e., skin conductance responses CS*+ > *CS–) has been previously established. Trait anxiety was not previously correlated with skin conductance responses in these datasets*.

**Q10: Please provide a brief timeline for the different steps in the preregistration.**

To maximize transparency, provide a brief time (e.g., in bullet points, a table) in which the (foreseen) date for the different steps in the planned research is specified. For example: *The syntax for the planned statistical analyses was finished on 15^th^ March 2019. An email will be sent to researchers X, Y, and Z on April 1^st^ 2019 and we will wait until May 31^st^ 2019 for a response. On July 1^st^ 2019 we will run the planned analyses*

## Discussion

In the current article we argue that it is possible and important to preregister analyses of preexisting data. We have presented several arguments for our position, considered the practical challenges, discussed several potential concerns, and proposed a template that researchers could use for preregistering analyses of preexisting data.

Increasingly efficient ways to gather, store, and analyze data creates more opportunity to test hypotheses on the basis of existing datasets. As we outlined above, there are many ways in which the evaluation of hypotheses can be biased when analyzing existing data (e.g., post-hoc hypothesizing). Preregistration can be a useful tool to prevent such biases. The template we have presented here is intended to provide general guidelines for the information which should be preregistered for analyses of preexisting data. We acknowledge, however, that the appropriateness of the template will of course depend on the specific features of each individual project. For some types of analyses of preexisting data (e.g., computational modelling) some questions in the template may be irrelevant (e.g., how the data will be obtained). We nonetheless encouraging researchers to use our template or other templates (e.g., Weston & Bakker, 2018) to provide maximal clarity about the steps in the research project to avoid potential biases.

We discussed many of the potential critiques against preregistration of analyses of preexisting data in the ‘Potential criticisms and concerns’ section. Even though preregistration can be challenging, requires time, and may be imperfect, it improves on earlier practices where transparency was lacking and reproducibility and replicability of research findings was problematic. Furthermore, as we discussed, most of the objections can be mitigated and preregistration may in fact be time-conserving rather than time-consuming for individual researchers and the discipline as a whole. Lastly, we strongly believe that the benefits of preregistration outweigh its costs as it helps to increase the trust in results by providing full transparency of the analysis plans, and can protect researchers against their own biases (Allen & Mehler, 2019; Lindsay, 2019; Wagenmakers & Dutilh, 2016). As such, we believe that the preregistration of analyses of preexisting data will improve the reliability, quality, and replicability of research findings in psychological research.

Finally, we acknowledge that our template for the preregistration of analyses of preexisting data is not completely fool-proof and that it is still possible for researchers to be insufficiently transparent about certain aspects of their research (for example not correctly reporting the exact date of data acquisition). However, transparency is not an all-or-nothing phenomenon. Progress will be necessarily incremental and the right incentives should be put in place by universities, funders, and journals to encourage researchers to be maximally transparent (Allen & Mehler, 2019). Our template is intended as a useful tool to facilitate such transparency.

## Footnotes

Note that replicability can be distinguished from reproducibility. As explained in the main text, replicability (also referred to as ‘results reproducibility’ and ‘experimental reproducibility’) refers to the repetition of a study’s results with newly collected data, using similar procedures as the original study. Reproducibility (or more precisely, ‘computational reproducibility’) refers to exactly reproducing the results of a study using the original data and data analysis steps of this study. Other types of reproducibility are ‘inferential reproducibility’ and ‘indirect reproducibility’ (which refer to reproducibility across analytical and experimental procedures). For a more extensive and fine grained discussion of the distinction between these different concepts see Goodman et al. (2016) and Leonelli (2018).
